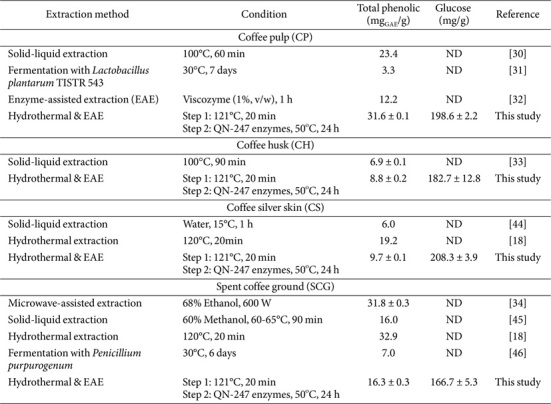# Mutant Strain of *Aspergillus aculeatinus* Boosts Total Phenolic Compounds and Sugar Recovery from Coffee Residues via Enzyme-Assisted Extraction

**DOI:** 10.4014/jmb.2412.12061

**Published:** 2025-06-12

**Authors:** Jantima Arnthong, Panida U-thai, Sa-ngapong Plupjeen, Piyada Bussadee, Wanlapa Lorliam, Sukhumaporn Krajangsang, Verawat Champreda, Surisa Suwannarangsee

**Affiliations:** 1 National Center for Genetic Engineering and Biotechnology (BIOTEC), National Science and Technology Development Agency (NSTDA), 113 Thailand Science Park, Klong Luang, Pathumthani 12120, Thailand; 2 Department of Microbiology, Faculty of Science, Srinakharinwirot University, 114 Sukhumvit 23, Wattana, Bangkok 10110, Thailand

**Keywords:** *Aspergillus aculeatinus*, cellulase, pectinase, coffee residues, polyphenols, enzyme-assisted extraction

## Abstract

Coffee residues are a valuable source of phenolic compounds and saccharides, which can be extracted through various methods such as solvent extraction, subcritical water extraction, and microwave-assisted extraction. Recently, enzyme-assisted extraction using microbial enzymes has emerged as a green and sustainable alternative. This study focused on enhancing cellulase and pectinase production in *Aspergillus aculeatinus* SF-034 through induced mutagenesis and evaluating the mutant enzymes for extracting polyphenols and saccharides from coffee by-products. The mutant QN-247 strain exhibited notably increased enzyme activities, with pectinase and CMCase levels approximately 31% and 120% higher than those of the mutant SF-034 and the wild-type strain, respectively. Scale-up in a 10-L bioreactor further confirmed high enzyme activities, reaching 995.8 U/ml for pectinase and 888.7 U/ml for CMCase. Enzyme-assisted extraction using the QN-247 mutant enzymes significantly enhanced the release of polyphenols (9.0-31.7 mg GAE/g) and glucose (166.7-208.3 mg/g biomass), outperforming commercial enzyme preparations under the assay conditions. These findings highlight the QN-247 strain as an efficient producer of multi-enzyme cocktails that offer a sustainable approach for extracting valuable bioactive compounds from coffee residues and other agricultural by-products.

## Introduction

Coffee is among the most widely consumed beverages worldwide due to its aromatic flavor and the health-promoting properties of caffeine and other bioactive compounds [1]. To date, the global coffee industry is projected to produce millions of tons of coffee annually, which reflects its significant demand and production scale [2]. However, the large-scale production of coffee also generates significant amounts of waste, primarily in the form of coffee residues including coffee husks, coffee pulps, spent coffee grounds, and coffee silver skins [3]. These pose environmental concerns due to the challenges associated with their disposal and management. Addressing the environmental impact of coffee residues is crucial as improper management can lead to soil and water pollution, greenhouse gas emissions, and the depletion of valuable resources [4].

The concept of utilizing agricultural wastes for the production of valuable compounds including polyphenols and saccharides has gained increasing attention in recent years [5]. In particular, coffee by-products are recognized as rich sources of phenolic compounds, primarily chlorogenic acid and its derivatives. These compounds possess antioxidant, anti-inflammatory, anticancer, and antimicrobial properties, making them highly valuable for applications in the pharmaceutical, cosmetic, and food industries [6]. Additionally, polysaccharides presented in coffee by-products such as cellulose, hemicellulose, and pectin can be hydrolyzed into oligosaccharides and monosaccharides that can be applied as prebiotics, sweeteners, and other functional ingredients [3].

Numerous studies have investigated the extraction of polyphenols and saccharides from various coffee residues using thermochemical techniques such as solvent extraction, subcritical water extraction, and microwave-assisted extraction [7, 8]. However, these methods present challenges including the potential degradation of heat-sensitive compounds, high energy demands, and concerns about environmental sustainability [6]. In response, recent research has focused on the use of microbial enzymes—specifically cellulases, hemicellulases, pectinases, and esterases—to degrade the complex plant material structures and release bioactive compounds through enzyme-assisted extraction. Given that plant bioactive compounds are associated with cell wall polysaccharides through hydrophobic interactions and hydrogen bonding, various hydrolytic enzymes such as cellulases and pectinases have been employed to cleave these bonds. These enzymes facilitate the degradation of structural polysaccharides within the distinct layers of the plant cell wall, thereby enhancing the extraction efficiency of phenolic compounds and carotenoids [9]. Cellulases specifically contribute to the degradation of cellulose, thereby loosening the structural integrity of the plant cell wall. Pectinases are responsible for the breakdown of pectic substances located in the middle lamella [10]. This enzymatic approach offers a promising alternative to conventional solvent-based methods by providing greater specificity, reduced energy consumption, and a more environmentally sustainable solution [11].

*Aspergillus aculeatinus* has demonstrated significant potential in producing a wide range of plant cell wall-degrading enzymes including cellulases (endoglucanases or carboxymethyl cellulase (CMCase) (EC 3.2.1.4), cellobiohydrolase (EC 3.2.1.91), and β-glucosidases (EC 3.2.1.21)), hemicellulases (xylanase (EC 3.2.1.8), β-xylosidase (EC 3.2.1.37), α-arabinofuranosidase (EC 3.2.1.55), pectinases (endo-polygalacturonase (EC 3.2.1.15), exo-polygalacturonase (EC 3.2.1.67), esterases, and lyases, which together constitute approximately 92% of its total secreted proteins [12]. Our previous study reported that *A. aculeatinus* SF-034 mutant strain achieved through a sequential mutagenesis approach produced about 64% and 63% higher levels of endoglucanase (CMCase) and pectinase, respectively, compared to its wild-type strain [13]. Despite these advancements, further development of hyper-producing strains is necessary to enable cost-effective enzyme production. Furthermore, the application of *A. aculeatinus* mutant enzymes for the extraction of polyphenols and saccharides from coffee residues remains underexplored.

This study aims to enhance the production of CMCase and pectinase in *A. aculeatinus* SF-034 through an induced mutagenesis using nitrous acid as a chemical mutagen. Subsequently, the effect of temperature and pH of these enzymes produced by the mutant strain were explored. The potential application of this mutant enzyme preparation in enzyme-assisted extraction processes for enhancing the recovery of polyphenols and saccharides from various coffee residues including coffee husks, coffee pulp, spent coffee grounds, and coffee silver skins was evaluated. The enzyme-assisted extraction approach by using *A. aculeatimus* mutant enzyme preparation developed in this study has the potential to contribute to the development of sustainable bioprocesses for the food, pharmaceutical, and cosmetic industries by valorizing agricultural waste.

## Materials and Methods

### Microorganism and Culture Conditions

The *A. aculeatinus* SF-034 mutant strain was obtained from our previous study [13] and was used as a parent strain for the generation of mutant strains exhibiting improved endoglucanase and pectinase activities. All fungal strains were cultured on potato dextrose agar (PDA) medium at 30°C and stored in 20% glycerol at −80°C.

### Raw Material Preparation

All coffee residues including coffee pulp (CP), coffee husk (CH), coffee silver skin (CS) and spent coffee ground (SCG) were kindly provided from Coffee innovation research unit, Srinakharinwirot University, Thailand. The coffee materials were dried at 70°C for 24 h in hot air oven. Subsequently, all samples underwent physical processing using a cutting mill (an Ultra Centrifugal Mill SM2000) equipped with a 0.5 cm mesh screen and were sieved to achieve particle sizes ranging from 180-425 μm (Retsch, Germany) and stored at room temperature until further study.

### Strain Improvement by Nitrous Acid Mutagenesis

The spore suspension of *A. aculeatinus* SF-034 was subjected to nitrous acid mutagenesis as previously described by [14]. The spores were suspended in citrate buffer at pH 3.0 and kept on ice. To initiate the nitrous acid treatment, 50 μl of 0.2 M sodium nitrite (NaNO_2_) was introduced into the suspension and was incubated at 30°C for 6 h. After that, the spores were thoroughly washed using a 0.2 M potassium phosphate (K_2_HPO_4_) solution. The treated spores were subsequently transferred into a mineral salt medium containing 1.86 g/l ammonium sulfate ((NH_4_)_2_SO_4_), 2 g/l potassium dihydrogen phosphate (KH_2_PO_4_), 0.3 g/l urea, 0.03 g/l calcium chloride dihydrate (CaCl_2_·2H_2_O), 0.3 g/l magnesium sulfate heptahydrate (MgSO_4_·7H_2_O), 4.08 g/l yeast extract, 5 mg/l ferrous sulfate heptahydrate (FeSO_4_·7H_2_O), 1.4 mg/l zinc sulfate heptahydrate (ZnSO_4_·7H_2_O), 2.0 mg/l cobalt chloride (CoCl_2_), 8.0 g/l peptone, 1.0 g/l Tween 80, and 10 g/l 2-deoxy-D-glucose (2-DG). The suspension was then incubated at 30°C with shaking at 200 rpm for 24 h. Following incubation, the germling spore solution was spread onto a mineral salt medium supplemented with 17 g/l agar and incubated at 30°C for 48 h to facilitate further growth.

The resulting mutants were subsequently screened for enhanced CMCase (endoglucanase) and pectinase production using agar plates containing 1% (w/v) carboxymethyl cellulose (CMC) or 1% (w/v) pectin as the sole carbon source, respectively. The cellulolytic activity of the mutants was evaluated based on the formation of a clear halo surrounding the fungal colonies. The enzymatic index (EI) was calculated as the diameter of the hydrolysis zone divided by the diameter of the colony [15]. Fungal colonies exhibiting an EI at least 5% greater than the wild-type strain were selected for further analysis. For pectinase activity, mutant strains exhibiting a colony diameter exceeding the wild-type strain by at least 5% were selected for further evaluation.

### Production of Cellulases and Pectinases

For enzyme production in flask scale, the spore suspension (1 × 10^7^ spore/ml) was inoculated into the production medium containing 1% (w/v) wheat bran and 0.5% (w/v) pomelo peel [13]. The culture was incubated at 30°C with shaking at 200 rpm for 6 days. The crude enzyme solution was collected after centrifugation at 9,016 ×g for 10 min at 4°C.

For large-scale enzyme production in a 10-L bioreactor, the sterilized production medium was inoculated with a spore suspension of *A. aculeatinus* QN-247 (1 × 10^9^ spores/ml). To prevent foaming during fermentation, 0.1%(v/v) of an antifoaming agent was added to the medium before autoclaving. Batch fermentation proceeded for 6 days at 30°C and 300 rpm without pH control. Aeration was maintained at a constant rate of 1 vvm. Samples were collected every 24 h throughout the fermentation process. After centrifugation, the supernatants were analyzed for CMCase and pectinase activities as well as total protein concentration. The experiment was conducted with two independent replicates.

### Enzymatic Activity Assays

Assay for CMCase or pectinase activities were performed using a reaction mixture containing 50 mM sodium acetate buffer (pH 5.0) and specific substrates: 1% (w/v) Carboxymethyl cellulose (CMC) or 0.5% (w/v) citrus pectin, respectively. All reactions were performed at 50°C for 10 min. The amount of reducing sugars released was quantified using the 3,5-dinitrosalicylic acid (DNS) method [16]. One unit (U) of EG or pectinase activity was defined as the amount of enzyme required to release 1 μmol of glucose or galacturonic acid per min under the assay conditions, respectively. Protein concentration in the crude enzyme solution was determined using Bradford's method [17] with Bio-Rad protein assay reagent (Bio-Rad, USA) and bovine serum albumin (BSA) as the standard protein. All assays were performed in triplicate.

### Characterization of *A. aculeatinus* QN-247 Enzyme Preparation

The optimum temperature of the crude enzyme from QN-247 strain was determined by incubating the mixture of the enzyme and 1% CMC in 50 mM sodium acetate buffer pH 5.0 for 10 min at different temperatures ranging from 30°C to 90°C. The thermostability of the enzyme was determined by incubating the enzyme in 50 mM sodium acetate buffer pH 5.0 for 60 min at temperatures ranging from 30°C to 90°C for 1 h.

The optimum pH of the mutant enzyme was determined by incubating the mixture of the enzyme and 1% CMC in the presence of buffers: 50 mM glycine-HCl (pH 3.0-3.5), 50 mM sodium acetate (pH 4.0- 6.0), 50 mM citrate phosphate (pH 6.5-7.0), 50 mM phosphate (pH 7.5-8.0) and 50 mM Tris-HCl (pH 8.5-9.0). The reaction mixtures were incubated at room temperature for 60 min. For the determination of pH stability, the enzyme was incubated in different buffers at different pH ranges (pH 3.0–9.0) for 10 min at 50°C.

To evaluate storage stability, the QN-247 enzyme preparation was stored at 4°C for 35 days. Enzyme samples were withdrawn at different time intervals to measure CMCase and pectinase activities. Additionally, a parallel experiment was conducted under the same conditions with the QN-247 enzyme preparation supplemented with 20% (w/v) PEG4000 as an additive. All enzymatic assays were performed in triplicate.

### LC-MS/MS Analysis of the QN-247 Crude Enzyme

For sample preparation, a total of 20 μg protein was reduced with 10 mM DTT in 10 mM ammonium bicarbonate at 62°C for 20 min, followed by alkylation at room temperature for 25 min in the dark. Samples were desalted using the Desalting column (Thermo Fisher Scientific, USA), then digested with trypsin (1:50, enzyme:protein) at 37°C for 6 h. Peptides were reconstituted in 0.1% formic acid and transferred to a TruView LCMS vial (Waters, UK).

Protein identification was performed using high-resolution Orbitrap mass spectrometry. Tryptic peptides were separated on an Ultimate 3000 LC system (Thermo Fisher Scientific) with an Easy-Spray C18 column. Mobile phases consisted of 0.1% formic acid in water (A) and 95% acetonitrile with 0.1% formic acid (B). Peptides were eluted with a 75-min linear gradient (5–45% B) at 300 μl/min. MS analysis employed a data-dependent TopN45 method with a higher-energy collisional dissociation at collision energy of 27, full scan range of m/z 350–1450, AGC target 300%, and resolution 180k; MS/MS scans were acquired at 15k resolution. Raw data were processed using Proteome Discoverer v2.4.2.0 against the *Aspergillus* database (retrieved 31 March 2025). Search parameters included: 10 ppm peptide mass tolerance, 0.05 Da fragment mass tolerance, trypsin digestion, fixed carbamidomethylation (C), variable oxidation (M), and a 1% false discovery rate (FDR) for peptide and protein identification.

### Enzyme-Assisted Extraction of Phenolic Compounds

A two-step extraction technique was employed to recover phenolic compounds from coffee residues. The first step involved hydrothermal pretreatment under mild conditions as previously described [18]. Briefly, 1 g of coffee residue was mixed with 20 ml of distilled water in a reaction vessel. The mixture was incubated at 121°C for 20 min and then were centrifuged at 10,414 ×g for 10 min to separate the solid and liquid fractions. The resulting supernatant was filtered through Whatman No. 4 filter paper and stored at -20°C in darkness until further analysis.

**Fig. 1:**
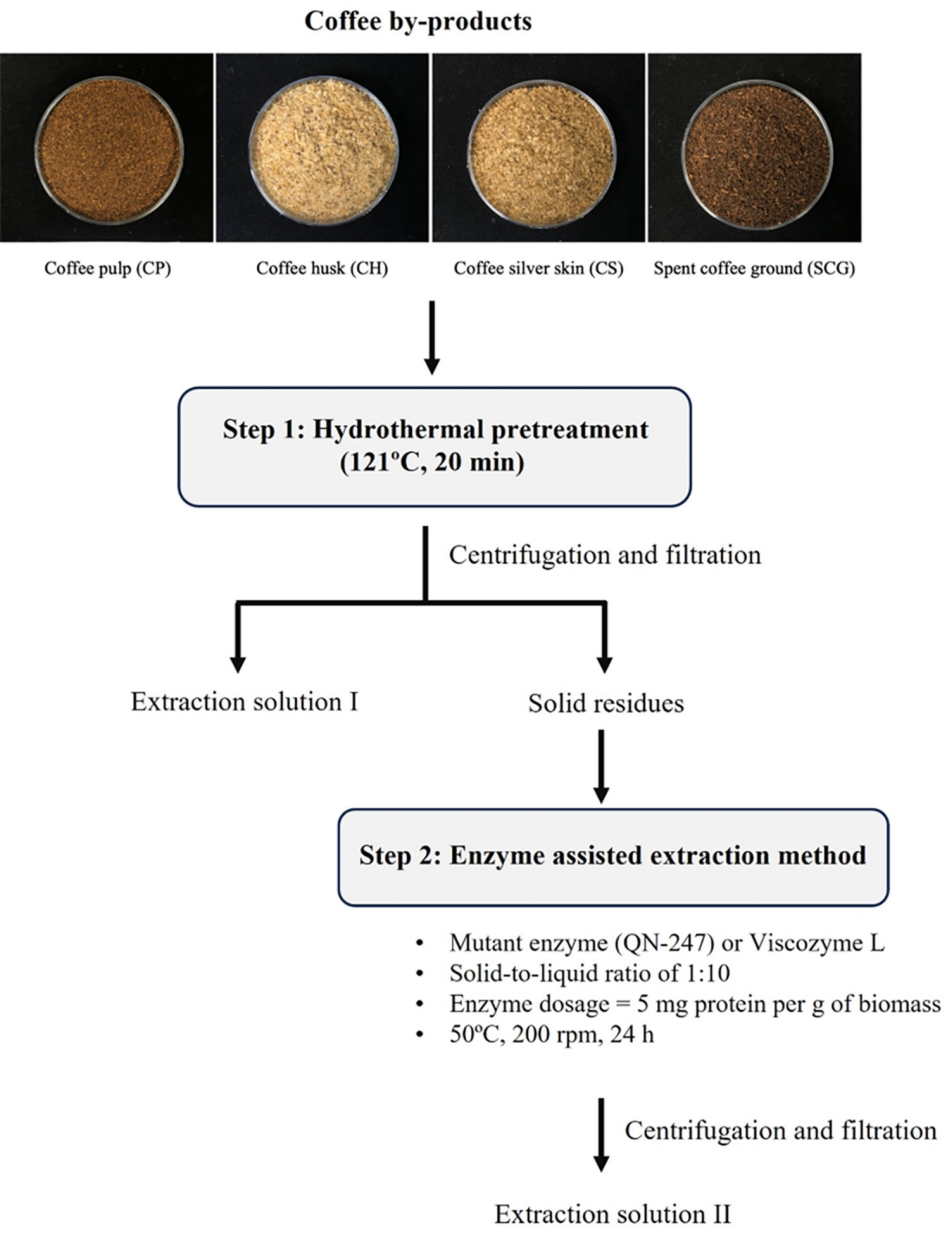
Process flow diagram for hydrothermal pretreatment followed by enzyme-assisted extraction of coffee by products.

Following the initial extraction of phenolic compounds, the remaining solid residues were subjected to enzyme-assisted extraction. This step compared the effectiveness of a mutant enzyme from *A. aculeatinus* QN-247 with a commercially available enzyme, Viscozyme L (Sigma, Germany). The reaction mixture contained coffee residue (solid-to-liquid ratio of 1:10) suspended in 50 mM sodium acetate buffer (pH 5.0) and supplemented with either the mutant enzyme or Viscozyme L at the same dosage of 5 mg total protein per gram of biomass. The mixture was incubated at 50°C with shaking at 200 rpm for 24 h. After incubation, the extraction solution was recovered by centrifugation and filtration. All experiments were performed in triplicate. The flowchart for phenolic compounds extraction from coffee residues was shown in Fig. 1.

### Determination of Total Phenolic and Total Reducing Sugar Content

The total phenolic content of the extracts was quantified using the Folin-Ciocalteu (FC) method as previously described [19]. Briefly, 0.5 ml of the extraction solution was mixed with 0.2 ml of FC reagent and incubated at room temperature for 10 min. Subsequently, the solution was neutralized with 0.6 ml of 20% sodium carbonate and incubated at 40°C for 30 min. The absorbance of the final solution was measured at 765 nm. Total phenolic content was expressed as milligram gallic acid equivalents per gram of dry weight biomass (mg GAE/g).

The concentration of monomeric sugars within the extract solution was determined using high-performance liquid chromatograph (HPLC) (Shmazu LC20, Japan) using Aminex HPX-87H column (Bio-Rad) with a column temperature of 65°C and flow rate of 0.5 ml/min of 5 mM H2SO4, equipped with a reflective index detector. All analyses were performed in triplicate.

### Statistical Analysis

The total phenolic content extracted from coffee by-products was statistically analyzed using one-way analysis of variance (ANOVA). The analysis was conducted in IBM SPSS Statistics software version 23 (SPSS Inc., USA) with a 95% confidence level (*p* < 0.05) to assess significant differences between treatments. Additionally, Tukey’s Honestly Significant Difference (HSD) post hoc test was applied to compare groups of samples for each parameter and allowed for the identification of statistically significant differences among the treatments. Where at least three replicates were applicable, the standard deviation (SD) of the data was calculated and presented as error bars in the figures.

## Results and Discussion

### Sequential Mutagenesis and Screening for CMCase and Pectinase Hyper-Producing Mutant

**Fig. 2:**
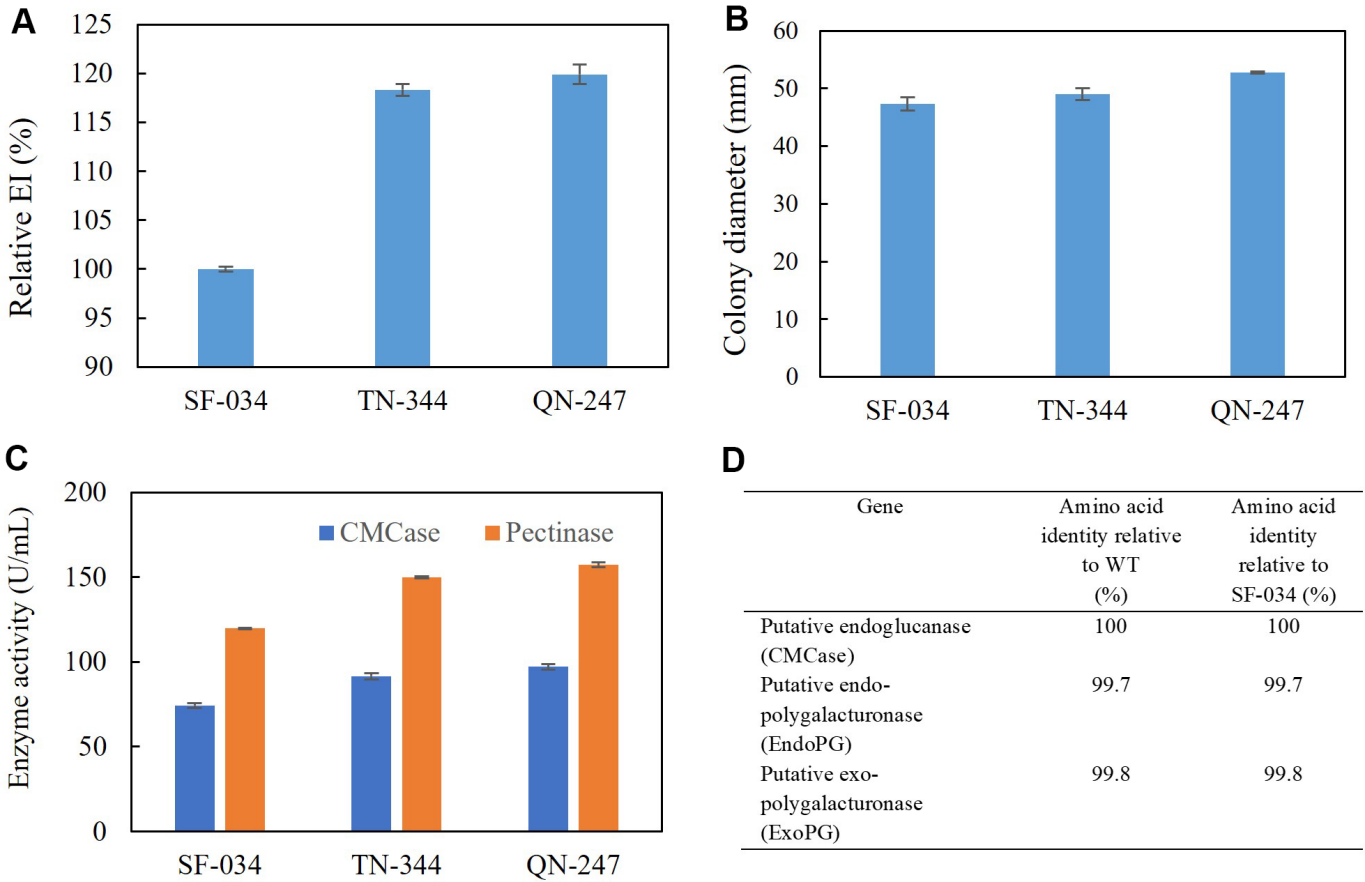
Comparison of agar plate screening and enzyme production between *A. aculeatinus* mutants, SF-034, TN-344, and QN247. (**A**) Relative Enzymatic Index (EI) in agar plate containing 1% CMC as sole carbon source. The relative EI of the SF-034 strain was defined as 100%. The experiments were performed using one biological sample. (**B**) Colony diameter on 1% pectin agar plate. The experiments were performed using one biological sample. (**C**) CMCase and pectinase activity produced by the mutant strain under shake flask cultivation. Data represent an average of three independent experiments. Error bars indicate SD. (**D**) Amino acid sequence identity (%) of selected cellulase and pectinase genes of QN-247 mutant relative to those of the wild-type and SF-034 double mutant strains.

In our previous study, the *A. aculeatinus* BCC60424 wild-type strain underwent sequential mutagenesis using ethyl methanesulfonate (EMS) and nitrous acid treatments [13]. The resulting SF-034 double mutant demonstrated a significant increase in CMCase and pectinase activities, exhibiting a 64% and 63% improvement, respectively, under shake flask cultivation. In the present study, the SF-034 strain was subjected to a third round of mutagenesis using nitrous acid to further enhance its enzyme production capacity. After screening the isolated mutants, strain TN-344 was identified as having the highest CMCase and pectinase activities and was selected for a fourth round of mutagenesis using the same approach. The quadruple mutant strain QN-247 exhibited the highest CMCase and pectinase activities, producing 157.3 ± 1.5 U/ml of pectinase and 97.1 ± 1.6 U/ml of CMCase, corresponding to 31% increases over the SF-034 strain. Fig. 2 provides a comparison of enzyme activity on agar plates and production levels among the *A. aculeatinus* mutants. Notably, TN-344 and QN-247 mutants showed increased enzyme index (EI) on CMC agar and greater colony diameters on pectin agar compared to strain SF-034. These results align with the enhanced CMCase and pectinase secretion observed in TN-344 and QN-247 mutants (Fig. 2C). Collectively, the quadruple mutant QN-247 demonstrated more than a 120% increase in enzyme secretion relative to the wild-type strain, highlighting its potential as a robust producer of endoglucanase and pectinase (Fig. S1).

To partially address the molecular basis underlying the enhanced enzyme production observed in the QN-247 mutant strain, DNA sequencing of selected cellulase and pectinase genes amplified from genomic DNA of the BCC60424 (WT), SF-034 double mutant, and QN-247 quadruple mutant strains were investigated. This analysis focused on one cellulase gene (putative endoglucanase) and two pectinase genes (putative endo-polygalacturonase and exo-polygalacturonase) (Fig. 2D). The amino acid sequence of the putative endoglucanase gene was found to be identical (100%) to that of its wild-type strain and the SF-034 mutant (Fig. S2), suggesting that this gene is conserved and unlikely to contribute to the enhanced phenotype. In contrast, for the pectinase genes, a single amino acid substitution was identified in both the endo- and exo-polygalacturonase genes of the QN-247 mutant strain (Figs. S3 and S4). These mutations may potentially influence protein structure or enzymatic function and thus could be associated with the improved enzyme activity observed [20]. Although these findings provide preliminary insight into genetic changes that may contribute to the QN-247 mutant phenotype, a more comprehensive investigation such as comparative whole-genome sequencing and transcriptomic analysis will be necessary to fully elucidate the molecular mechanisms responsible for enhanced enzyme production [21].

Furthermore, the enzyme activity profile of the crude enzyme produced by QN-247 mutant revealed the presence of a broad range of biomass-degrading enzymes including cellulases (FPase, CMCase, BGL), pectinases (pectinase, endo-polygalacturonase), xylanase, mannanase, amylase, and β-glucanase (Table S2). Compared to the wild-type and SF-034 double mutant strains, the QN-247 exhibited higher total protein secretion (1.59 ± 0.03 g/l) than the SF-034 (0.97 ± 0.02 g/l) and WT (0.73 ± 0.01 g/l). This increase correlated with enhanced volumetric activities of pectinase (2.2 times higher than WT; 1.3 times higher than SF-034) and CMCase (2.0 times higher than WT; 1.2 times higher than SF-034). Notably, endo-polygalacturonase activity, one of a key pectinase, was enhanced by 2.4-fold compared to the SF-034 strain. However, specific activities of cellulases, pectinases, and other hydrolytic enzymes in the QN-247 crude enzyme showed a slight decline, likely due to the increased total protein content. This evidence supports our interpretation that the enhanced performance of the QN-247 mutant may be associated with increased enzyme production and/or secretion efficiency. To further validate this observation, a quantitative proteomic analysis or zymogram-based approach comparing QN-247 and its parental strains would provide valuable insight into changes in the abundance and activity of specific secreted enzymes, thereby helping to elucidate the molecular basis of the enhanced enzymatic performance.

### Enzyme Characterization of *A. aculeatinus* QN-247 Mutant

**Fig. 3:**
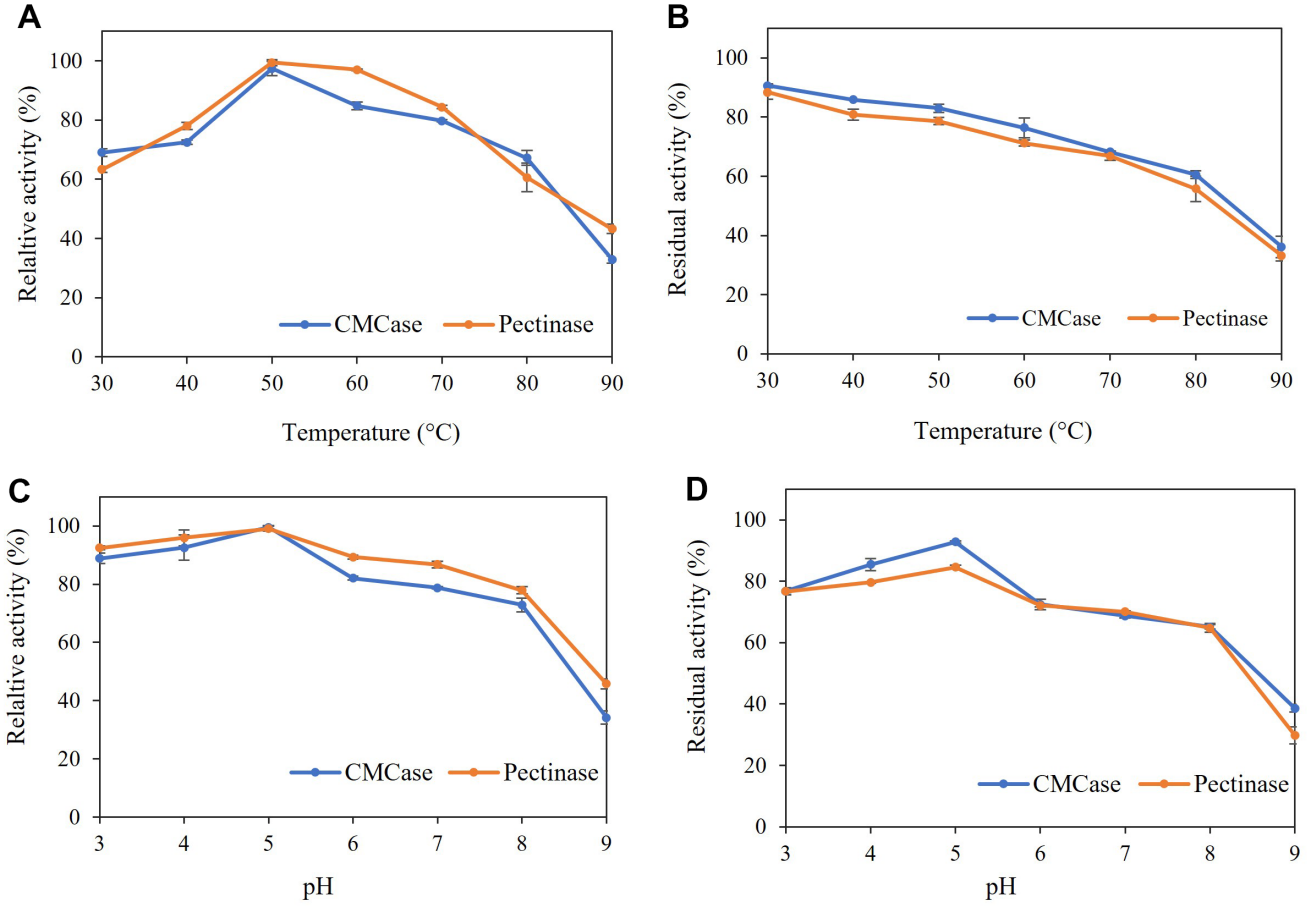
Characteristics of CMCase and pectinase produced by *A. aculeatinus* QN-247. (**A**) Effect of temperature. (**B**) Thermal stability. (**C**) Effect of pH. (**D**) pH stability. Data represent an average of three independent experiments. Error bars indicate SD.

To determine the optimum temperature and pH for the enzyme preparation from *A. aculeatinus* QN-247, we measured the activities of CMCase and pectinase under various temperature and pH conditions. Our results indicate that CMCase and pectinase activity peaked at 50°C (Fig. 3A). Temperatures exceeding 60°C resulted in a decrease in the activities of both enzymes. In terms of thermal stability, both CMCase and pectinase retained over 70% of their initial activities after being incubated at temperatures ranging from 30 to 60°C for 1 h prior proceed the enzyme activity assay at 50°C (Fig. 3B). Conversely, incubation at 80 to 90°C for the same duration led to a loss of more than 40% of their initial activity. Fig. 3C illustrates the effects of pH on CMCase and pectinase activities, revealing that the optimal pH for both enzymes is 5.0. Furthermore, the enzyme preparation from *A. aculeatinus* QN-247 demonstrated robust activity across a wide pH range of 3.0 to 7.0. However, pH stability assays indicated a decline in enzyme activity after 1 h of incubation at pH levels between 6.0 and 9.0 (Fig. 3D). These findings align with previous reports indicating optimal temperatures for CMCase and pectinase activities at 50°C, as well as an optimal pH around 5.0, consistent with other *Aspergillus* cellulases and pectinases [22, 23]. The similarity in operational ranges for cellulases and pectinases suggests potential advantages for enzyme-assisted extraction processes, particularly for simultaneously deconstruction of polysaccharide and the release of polyphenolic compounds [11]. Additionally, the purification and characterization of individual CMCase and pectinase enzymes should be conducted in future studies to gain deeper insight into their substrate specificity and hydrolysis products.

**Fig. 4:**
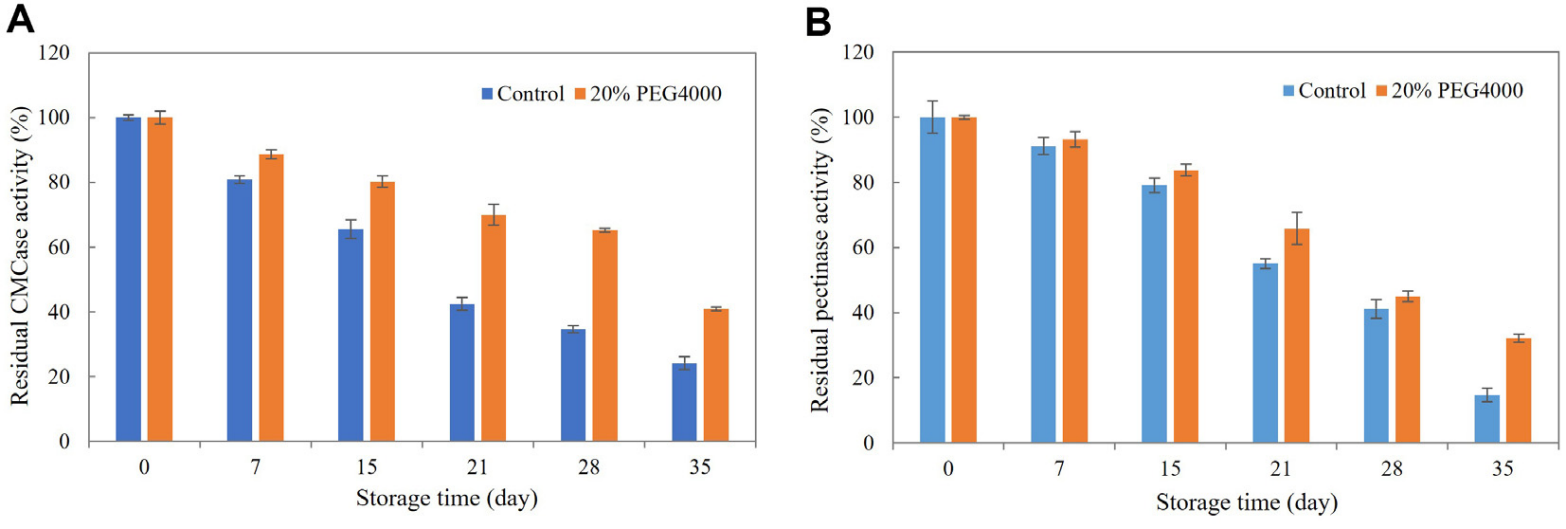
Storage stability of crude enzyme preparations from *A. aculeatinus* QN-247 in the absence (control) and presence of 20% (w/v) PEG4000. Data represent the average of three independent experiments. Error bars indicate SD.

Since long-term enzyme storage stability is crucial for industrial applications, the storage stability of the QN-247 enzyme preparation was evaluated with and without 20% (w/v) PEG4000 supplementation. After storage at 4°C, the residual CMCase and pectinase activity were measured to assess their stability over time. As shown in Fig. 4, CMCase activity retained 65.5% of its initial activity after 15 days of storage, but declined to less than 50%after 21 days at 4°C. In contrast, the addition of PEG4000 significantly improved CMCase stability by maintaining more than 80% of its initial activity after 15 days of storage. A similar trend was observed for pectinase activity. In the absence of PEG4000, pectinase retained 79.1% of its initial activity after 15 days, but declined to less than 50%after 28 days at 4°C. However, supplementation with 20% (w/v) PEG4000 further enhanced stability by preserving up to 83.8% of its initial activity after 15 days of storage. The use of additives is widely recognized as an effective strategy for enhancing enzyme storage stability [24]. Studies on the activation and stabilization mechanisms of ten starch-degrading enzymes have suggested that various additives including polyethylene glycols (PEGs), interact with enzyme-proteins to promote an optimally folded and compact barrel motif structure. This structural conformation is believed to maximize enzymatic activity and stability by preventing denaturation and preserving functional integrity during storage [25].

### Protein Identification of Crude Enzyme from *A. aculeatinus* QN-247 Mutant

To determine the enzyme composition of the secreted proteins from the *A. aculeatinus* QN-247 mutant, crude enzyme preparations were analyzed using LC-MS/MS based proteomic analysis. Protein identification was performed using Proteome Discoverer v2.4.2.0, referencing the *Aspergillus* database. As shown in Table 1, a total of 12 individual proteins were identified. Notably, five of these were classified as pectinases including endo-polygalacturonase (GH28), probable pectin lyase A (PL1), pectin lyase (PL1), probable endopolygalacturonase E (GH28), and pectin esterase (PE). Additionally, one cellulase (GH5) and two hemicellulases (arabinogalactan endo-β-1,4-galactanase (GH53) and probable endo-1,4-β-xylanase C (GH10)) were detected. These findings are consistent with previous reports on *A. aculeatinus* strain O822, in which pectinases, cellulases, and hemicellulases constituted the major secreted enzymes [12]. However, several enzymes such as cellulose 1,4-β-cellobiosidase (CBH), glucan 1,4-β-glucosidase (BGL), and rhamnogalacturonan hydrolases, were not detected in this study. This absence may be attributed to differences in culture conditions or limitations related to the detection threshold of the LC-MS/MS analysis. Nevertheless, the presence of cellulase activities (FPase, CMCase, and BGL) in the crude enzyme activity assays supports the existence of these enzyme classes in the *A. aculeatinus* QN-247 mutant.

### Scale-Up of Concurrent CMCase and Pectinase Production of *A. aculeatinus* QN-247 in a 10-L Bioreactor

**Fig. 5:**
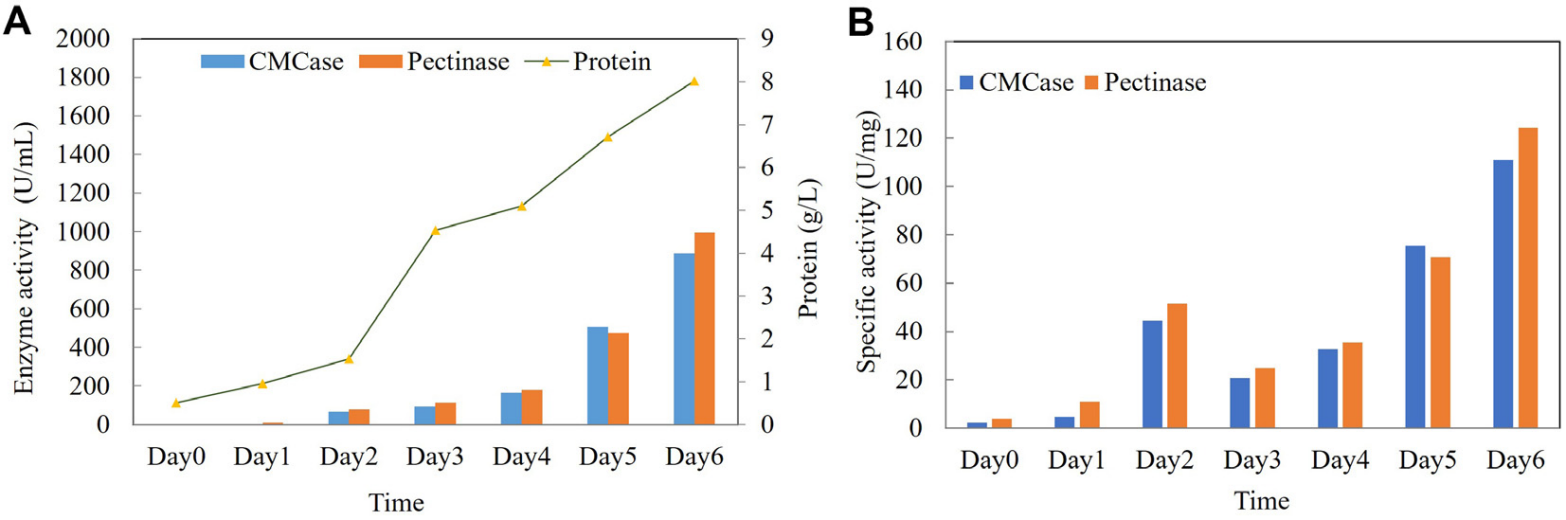
Enzyme activity and protein content produced by *A. aculeatinus* QN-247 mutant strain under submerged fermentation in a 10-L bioreactor. (**A**) Volumetric activity (U/ml). (**B**) Specific activity (U/ mg protein). Data represent an average of two independent batches.

The production of enzymes by *A. aculeatinus* QN-247 was carried out in a 10-L bioreactor, utilizing the same enzyme production medium as employed in shake flask cultivation. Based on our previous experiments, enzyme production by the *A. aculeatinus* double mutant (SF-034) was monitored for 7 days. The results indicated that CMCase and pectinase activities increased throughout the cultivation period and reached their maximum levels on day 6, followed by a subsequent decline [13]. Therefore, in this study, enzyme production in a 10-L bioreactor was conducted for 6 days to align with the previous findings. As shown in Fig. 5, both the total secreted protein and enzyme activities exhibited an upward trend over the cultivation period. After cultivation for 6 days, the CMCase activity reached 888.7 U/ml (111.0 U/mg protein), while the pectinase activity was detected at 995.8 U/ml (124.2 U/mg protein). Additionally, the total secreted protein concentration increased up to 8.0 g/l. These values represent a substantial increase in enzyme production yields compared to those achieved in 250-ml shake flask fermentations. This improvement underscores the benefits of submerged fermentation (SmF) in bioreactors, which provide a controlled environment with continuous air supply, thereby enhancing enzyme productivity [26]. Additionally, this represents improvements of 52.4% and 102.3% in CMCase and pectinase activities, respectively, compared to the previously published SF-034 strain [13].

While numerous studies have highlighted the potential of solid-state fermentation (SSF) as an alternative cultivation method for cellulase and pectinase production, SmF remains the most favorable system for large-scale enzyme production as it allows for better control of environmental parameters due to its scalability, better control of environmental parameters, and efficiency in downstream processing [27]. In comparison to earlier reports, *A. niger* produced pectinase activity of 450 U/ml under fed-batch conditions in a 16-L bioreactor [27], which is slightly lower than the performance observed for our QN-247 mutant (Table 2). Another study reported that the CMCase production by *Penicillium occitanis* Pol6 and *Streptomyces* sp. T301 under submerged fermentation was reported to be 21 U/ml [28] and 148 U/ml [29], respectively, which is lower than the yield achieved by the QN-247 mutant strain in this study. However, differences in medium composition and bioprocess parameters may influence enzyme production levels and should be taken into account when making such comparisons.

In this study, the *A. aculeatinus* QN-247 mutant strain exhibited high production titers of both CMCase and pectinase, highlighting its potential for the concurrent production of these enzymes in industrial applications. To further enhance its utility, future research could focus on optimizing fermentation parameters such as carbon-to-nitrogen ratio, pH, and aeration to maximize enzyme yields. In addition, a comprehensive techno-economic analysis will be essential to evaluate the feasibility and cost-effectiveness of deploying this strain for large-scale enzyme production.

### Enzyme-Assisted Extraction of Polyphenol and Sugars from Coffee By-Products

To evaluate the potential of crude enzyme preparations from the *A. aculeatinus* QN-247 mutant for enzyme-assisted extraction of polyphenols and saccharides from coffee by-products, four major coffee processing residues were collected: coffee pulp (CP), coffee husk (CH), coffee silver skin (CS), and spent coffee grounds (SCG). These by-products are known to contain substantial amounts of carbohydrates (57-85%), cellulose (12-43%), hemicellulose (13-39%), lignin (23-28%), pectin (6.5-20%), and protein (8-19%) as detailed in Table S1. In this study, all coffee by-products underwent hydrothermal pretreatment followed by enzyme-assisted extraction using the crude QN-247 enzyme. The extraction was performed at 50°C, pH 5.0 for 2 h. To compare the performance of the QN-247 enzyme, a commercial cellulase and pectinase preparation (Viscozyme L) was used under the same conditions. The soluble fractions from each step were collected for analysis of total phenolic compounds and saccharides.

**Fig. 6:**
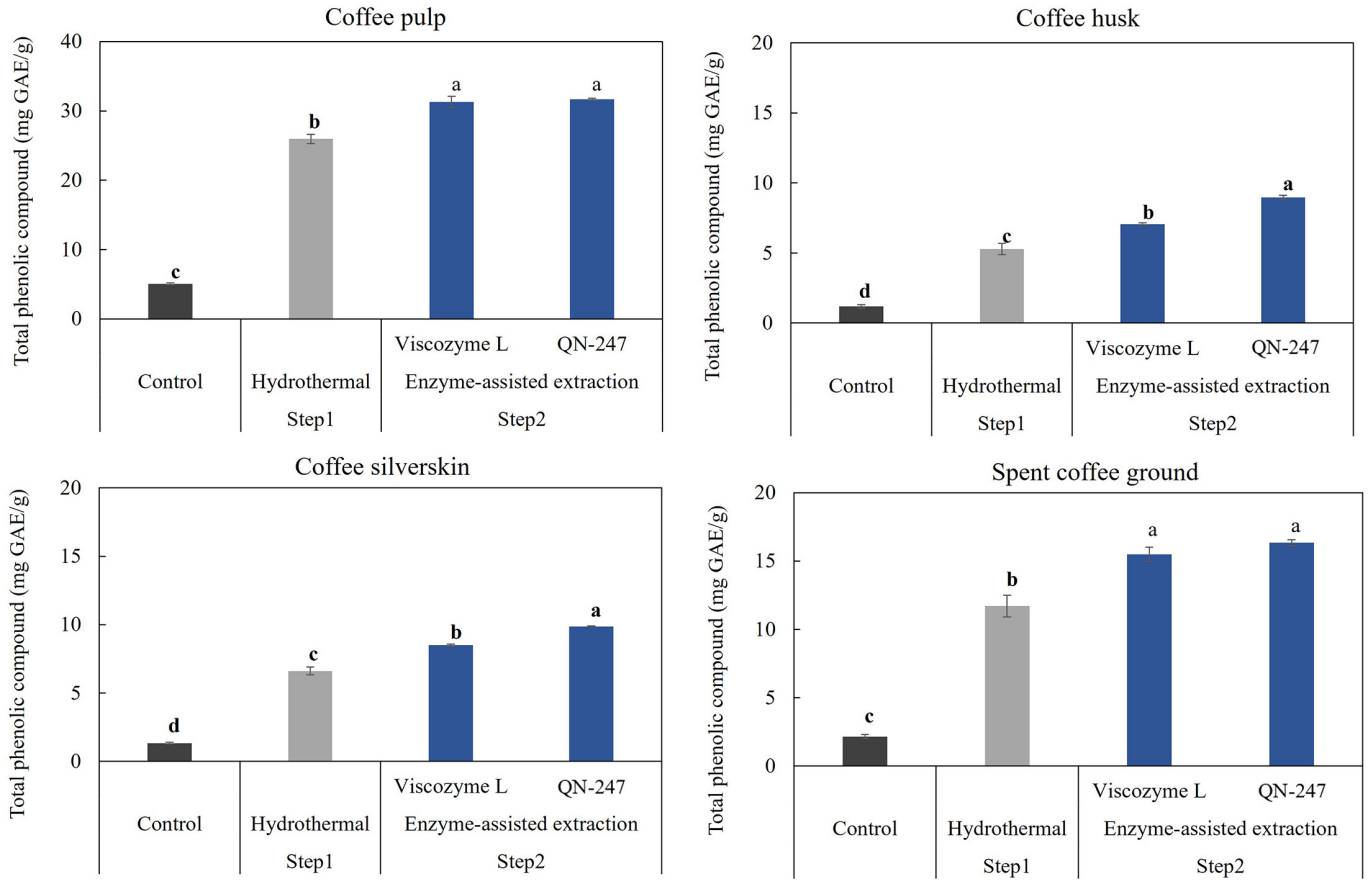
Total phenolic compounds content extracted from coffee-by products by hydrothermal pretreatment followed by enzyme-assisted extraction using *A. aculeatinus* QN-247 mutant enzyme and Viscozyme L. Data represent an average of three independent experiments and were analyzed using one-way ANOVA followed with Tukey’s HSD (Honestly Significant Difference) test (*p*<0.05). Error bars indicate SD.

**Total phenolic content (TPC).** The TPC in the soluble fraction of coffee by-products was quantified using the Folin-Ciocalteu method (Fig. 6). The results revealed a significant increase in TPC across all coffee residue extracts following hydrothermal pretreatment coupled with enzyme-assisted extraction. Notably, the extracts of CH and CS treated with the QN-247 enzyme exhibited significantly higher TPC compared to those extracted with a commercial enzyme (Viscozyme L) at the same dosage (*p* < 0.05). This result suggests that the QN-247 enzyme profile might be more suitable for breaking down the structure of CH and CS than that of the Viscozyme L. Further investigation is required to elucidate the mechanisms by which these enzymes enhance phenolic compound release. Additionally, TPC content varied among different coffee residue types. CP extract exhibited the highest TPC, reaching 31.6 ± 0.1 mg gallic acid equivalents (GAE) per g biomass, followed by SCG extract, which contained 16.3 ± 0.3 mg GAE/g. These values represent a 6.3-fold and 7.6-fold increase over the untreated control samples, respectively.

In comparison, the TPC of CP extract obtained through hydrothermal treatment followed by enzyme-assisted extraction using QN-247 mutant enzymes was substantially higher compared to other methods (Table 3). These include solid-liquid extraction (23.4 mg GAE/g) [30], fermentation with *Lactobacillus plantarum* TISTR 543 (3.3 mg GAE/g) [31], and enzyme-assisted extraction using 1% Viscozyme (12.2 mg GAE/g) [32]. For CH, a previous study by Rebollo-Hernanz *et al*. [33] reported a maximum TPC of 6.9 mg GAE/g from hydrothermal treatment at 100°C for 90 min, which is lower than the values observed in this study (8.8 ± 0.2 mg GAE/g). However, the TPC of CS and SCG extracts obtained in this study was lower than those reported using hydrothermal extraction at 120°C for 20 min [18] or microwave-assisted extraction with ethanol [34]. Variations in phenolic content may result from differences in geographic origin, types of coffee residues, and processing methods [35]. Therefore, the high TPC observed in CP and CH extracts highlights the effectiveness of the QN-247 enzyme in enzyme-assisted extraction, offering a sustainable and efficient method for maximizing the recovery of phenolic compounds.

**Fig. 7:**
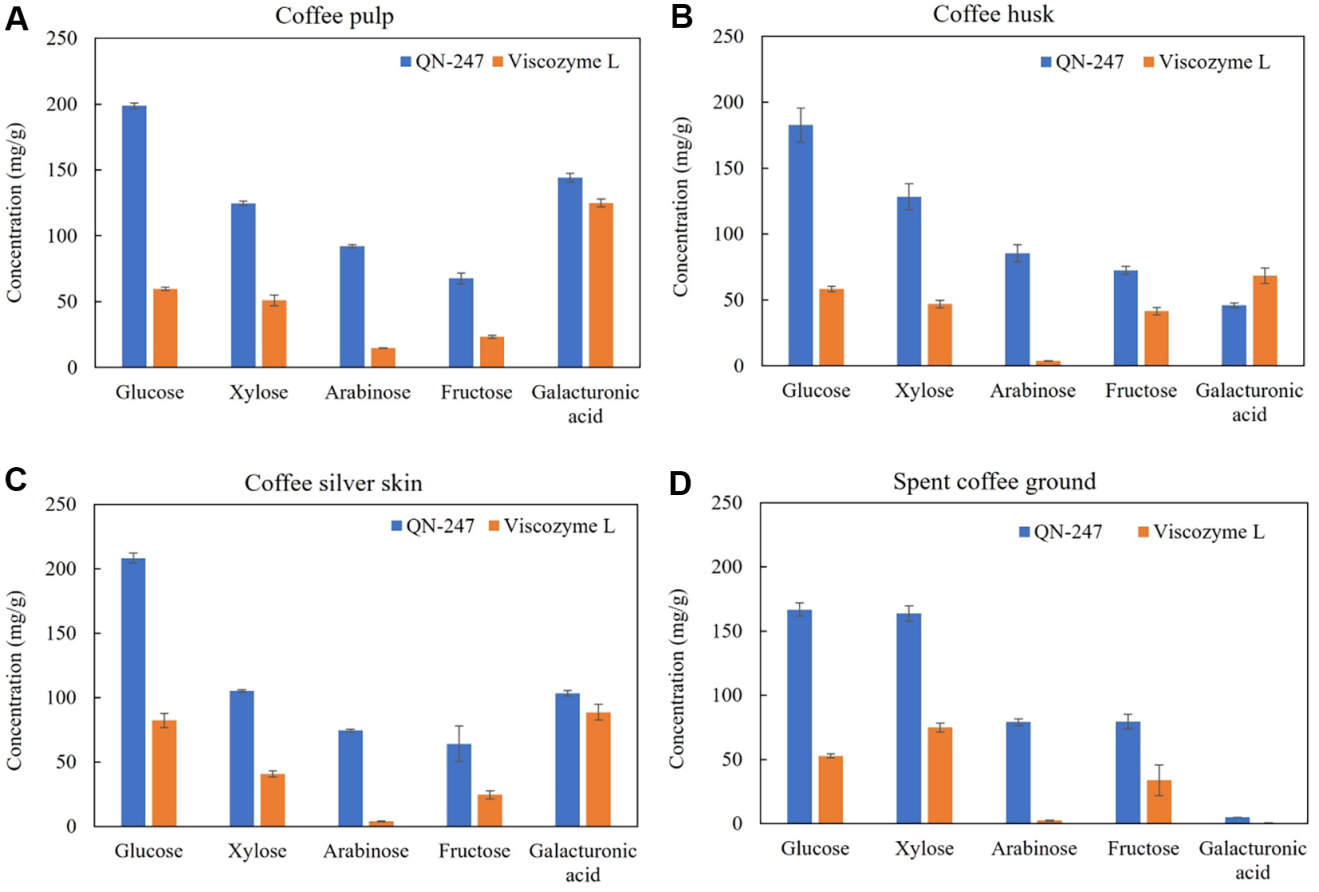
Sugar concentration extracted from coffee-by products by hydrothermal pretreatment followed by enzyme-assisted extraction using *A. aculeatinus* QN-247 mutant enzyme and Viscozyme L. Data represent an average of three independent experiments. Error bars indicate SD.

**Sugars.** Polysaccharides such as cellulose, hemicellulose, and pectin in coffee residues can be efficiently broken down into smaller saccharide molecules through treatment with hydrolytic enzymes [36, 37]. In this study, the monomeric sugar content in the soluble fractions extracted from coffee by-products via enzyme-assisted extraction was analyzed using HPLC (Fig. 7). The results showed that glucose was the predominant monomeric sugar released through enzyme-assisted extraction across all coffee residues, consistent with the previous observation on coffee powder waste [38]. The concentrations of other sugars derived from hemicellulose and pectin hydrolysis such as xylose, fructose, arabinose, and D-galacturonic acid varied depending on the type of coffee residues. The highest glucose content was observed in CS (208.3 ± 3.9 mg/g biomass) and CP (198.6 ± 2.2 mg/g biomass) when treated with the QN-247 enzyme, representing 2.5-fold and 3.3-fold increases, respectively, compared to Viscozyme L. These soluble sugars extracted from coffee residues can serve as carbon sources for fermentation or as raw materials for further conversion into high-value bioproducts [39]. The findings demonstrate the efficiency of the QN-247 enzyme preparation in sugar recovery and highlight its potential for enzyme-assisted extraction processes.

Based on the LC-MS/MS proteomic analysis of the QN-247 crude enzyme preparation, we identified a diverse set of biomass-degrading enzymes including cellulases, pectinases (polygalacturonases, pectin lyases, pectin esterase), as well as accessory enzymes such as xylanases, amylase, and mannanase. When compared with previously published proteomic data for Viscozyme L [40], we found that the key cellulase and pectinase families present in QN-247 were largely consistent with those reported for Viscozyme L. This suggests a functional similarity between the two enzyme systems, both of which are derived from *Aspergillus*-based production platforms. However, it is important to note that Viscozyme L has been reported to contain over 150 individual proteins, whereas our LC-MS/MS analysis identified 12 distinct proteins in the QN-247 enzyme preparation. This discrepancy is likely attributable to differences in cultivation conditions, sample preparation protocols, and particularly the analytical criteria used in proteomic analysis such as peptide detection sensitivity and database matching stringency. Therefore, while the core enzyme activities appear comparable, differences in enzyme abundance and substrate specificity may contribute to the variations in sugar composition observed during enzyme-assisted extraction.

In this study, the *A. aculeatinus* mutant QN-247 with enhanced CMCase and pectinase secretion was successfully developed through multiple rounds of induced mutagenesis. The mutant strain exhibited significantly increased CMCase and pectinase activities, achieving up to 120% higher levels compared to the wild-type strain. Submerged fermentation in a 10-L bioreactor further enhanced enzyme production, demonstrating the robustness of the mutant strain. The crude enzyme preparation from *A. aculeatinus* mutant QN-247 showed excellent performance in enzyme-assisted extraction of phenolic compounds and sugars from coffee by-products. Phenolic compounds, known for their antioxidant properties and associated health benefits, and sugars like glucose and fructose, which can serve as substrates for various microbes, make these coffee residue extracts valuable for applications in the food, pharmaceutical, and cosmetic industries. It has been reported that chlorogenic acid and its derivatives are the predominant phenolic compounds found in coffee residues [41]. However, to obtain a more comprehensive profile of the phenolic compounds and other bioactive substances extracted using the mutant enzyme, advanced analytical techniques such as HPLC [42] or UPLC-MS/MS [43] should be utilized in future studies. Furthermore, a systematic optimization of key process parameters including enzyme dosage, extraction time, temperature, and pH would be further enhance extraction efficiency. Future research will address these aspects to establish the most effective enzyme-assisted extraction conditions for maximizing phenolic compound and sugar recovery. Therefore, this study highlights the advantages of using *A. aculeatinus* mutant QN-247 enzymes for valorizing bioactive compounds in agricultural residues, providing a more efficient and sustainable alternative to conventional extraction methods.

## Supplemental Materials

Supplementary data for this paper are available on-line only at http://jmb.or.kr.



## Figures and Tables

**Table 1:** Protein identification of crude enzyme preparation from *A. aculeatinus* QN-247 mutant using LCMS/MS proteomic analysis.

UniProKB Accession	Description	Coverage (%)	No. of Peptides	No. of PSMs^1^	Amino acids	Protein family^2^
A0A117E3H6	Glucoamylase	14	4	18	643	GH15
A0A100I2I9	Transcription factor AbaA	10	3	5	831	-
A0A124BXT4	endo-polygalacturonase	11	3	11	370	GH28
A0A124BXE9	Acid-stable alpha-amylase	7	3	5	1181	-
C5J411	Probable endo-1,4-beta-xylanase C	12	3	6	327	GH10
A2R3I1	Probable pectin lyase A	15	3	19	379	PL1
A0A3F3RCS1	Salicylate synthase	4	3	11	2210	-
G3YEH1	Arabinogalactan endo-beta-1,4-galactanase	16	3	8	350	GH53
A0A100IK89	pectin lyase	17	3	20	379	PL1
A0A100ILF3	cellulase	12	3	6	534	GH5
A2QBB6	Probable endopolygalacturonase E	13	3	19	378	GH28
A0A100IC32	Pectinesterase	15	3	8	327	PE

^1^PSMs = peptide/spectrum matches

^2^GH = Glycoside Hydrolase; PL = Polysaccharide Lyase; PE = Pectin esterase

**Table 2:** Comparison of CMCase and pectinase activities of *A. aculeatinus* QN-247 and other microbial strains.

Microorganisms	Enzyme activity (U/ml)		Type and scale of fermentation	References
	CMCase (U/ml)	Pectinase (U/ml)		
*A. aculeatinus* QN-247	888.7	995.8	SmF, 10-Lbioreactor	This study
*A. aculeatinus* SF-034	583.3	492.3	SmF, 10-L bioreactor	[13]
*Aspergillus niger*	-	109.6 U/ml	SmF, Batch, 16-L bioreactor	[27]
		450 U/ml	SmF, Fed-batch, 16-L bioreactor	
*Penicillium occitanis* Pol6	21	-	SmF, Fed-batch, 20-L bioreactor	[28]
*Streptomyces* sp. T3-1	148	-	SmF, Batch, 50-L bioreactor	[29]

**Table 3:** Extraction methods for phenolic compound and sugar extraction from different coffee by-products.