# Supplementary material for: Mutant Strain of Aspergillus aculeatinus Boosts Total Phenolic Compounds and Sugar Recovery from Coffee Residues via Enzyme-Assisted Extraction
Source: J Microbiol Biotechnol. 2025 Jun 12;35:e2412061. doi: 10.4014/jmb.2412.12061 (PMC12197818; doi:10.4014/jmb.2412.12061)
Supplement: Supplementary file 1 [file jmb-35-e2412061-supple.pdf]

Supporting Information

Mutant strain of *Aspergillus aculeatinus* boosts total phenolic compounds and sugar recovery from coffee residues via enzyme-assisted extraction

Jantima Arnthong[1#], Panida U-thai[1#], Sa-ngapong Plupjeen[1], Piyada Bussadee[1], Wanlapa Lorliam[2], Sukhumaporn Krajangsang[2], Verawat Champreda[1], Surisa Suwannarangsee[1*]

[1] National Center for Genetic Engineering and Biotechnology (BIOTEC), National Science and Technology Development Agency (NSTDA), 113 Thailand Science Park, Klong Luang, Pathumthani 12120, Thailand

[2] Department of Microbiology, Faculty of Science, Srinakharinwirot University, 114 Sukhumvit 23, Wattana, Bangkok 10110, Thailand.

[#]These authors contributed equally to this work

*** Correspondence:** Dr. Surisa Suwannarangsee

Phone: +6625646700, Fax: (66) 2564 6707, Email: surisa.suw@biotec.or.th

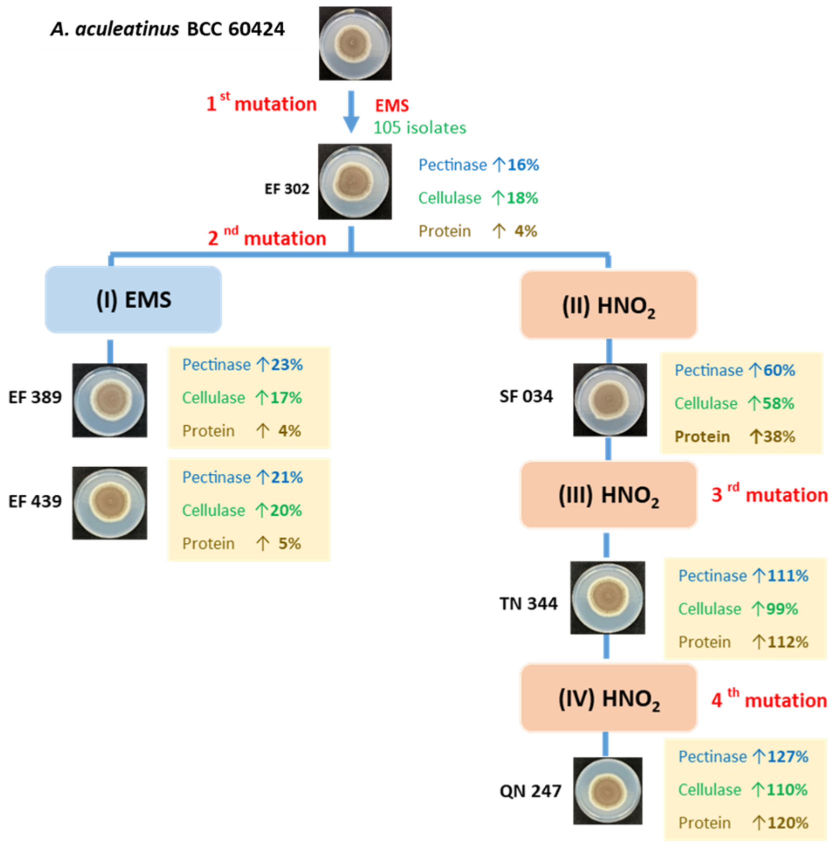

Figure S1. Pedigree of *Aspergillus aculeatinus* mutant strains and their cellulase (CMCase) and pectinase production capacities developed in [13] and in the present study.

Putative endoglucanase

```
EG_WT           MKLLNLLVAAAAAGSAVAAPTHEHTKRASVFEWIGSNESGAEFGTAIPGTWGIDYIFPDT
EG_SF           MKLLNLLVAAAAAGSAVAAPTHEHTKRASVFEWIGSNESGAEFGTAIPGTWGIDYIFPDT
EG_QN           MKLLNLLVAAAAAGSAVAAPTHEHTKRASVFEWIGSNESGAEFGTAIPGTWGIDYIFPDT
                ************************************************************

EG_WT           SAIATLVSKGMNIFRVQFMMERLVPNSMTGSYDEAYLNNLTTVVNAITAAGVHAIVDPHN
EG_SF           SAIATLVSKGMNIFRVQFMMERLVPNSMTGSYDEAYLNNLTTVVNAITAAGVHAIVDPHN
EG_QN           SAIATLVSKGMNIFRVQFMMERLVPNSMTGSYDEAYLNNLTTVVNAITAAGVHAIVDPHN
                ************************************************************

EG_WT           YGRYNNEIISSTADFQTFWQNLAGQFKDNDLVIFDTNNEYNTMDQTLVLDLNQAAIDGIR
EG_SF           YGRYNNEIISSTADFQTFWQNLAGQFKDNDLVIFDTNNEYNTMDQTLVLDLNQAAIDGIR
EG_QN           YGRYNNEIISSTADFQTFWQNLAGQFKDNDLVIFDTNNEYNTMDQTLVLDLNQAAIDGIR
                ************************************************************

EG_WT           AAGATSQYIFAEGNSWSGAWTWADINDNMKALTDPQDKLVYEMHQYLDSDGSGTSGVCVS
EG_SF           AAGATSQYIFAEGNSWSGAWTWADINDNMKALTDPQDKLVYEMHQYLDSDGSGTSGVCVS
EG_QN           AAGATSQYIFAEGNSWSGAWTWADINDNMKALTDPQDKLVYEMHQYLDSDGSGTSGVCVS
                ************************************************************

EG_WT           ETIGAERLQAATQWLKDNGKVGILGEYAGGANDVCRTAIAGMLEYMANNTDVWKGAVWWA
EG_SF           ETIGAERLQAATQWLKDNGKVGILGEYAGGANDVCRTAIAGMLEYMANNTDVWKGAVWWA
EG_QN           ETIGAERLQAATQWLKDNGKVGILGEYAGGANDVCRTAIAGMLEYMANNTDVWKGAVWWA
                ************************************************************

EG_WT           AGPWWADYMFSMEPPSGPAYSGMLDVLEPYLGW
EG_SF           AGPWWADYMFSMEPPSGPAYSGMLDVLEPYLGW
EG_QN           AGPWWADYMFSMEPPSGPAYSGMLDVLEPYLGW
                *********************************
```

Figure S2. Amino acid sequence alignment of putative endoglucanase gene from BCC60424 (WT), SF-034 double mutant (SF), and QN-247 quadruple mutant (QN) strains.

Putative endopolygalacturonase

```
Endo_WT          MHSFQLLGLAAVGSVVSAAPTASRVSDLVKKSSSTCTFTSASEASESISSCSNVVLSNIE
Endo_SF          MHSFQLLGLAAVGSVVSAAPTASRVSDLVKKSSSTCTFTSASEASESISSCSNVVLSNIE
Endo_QN          MHSFQLLGLAAVGSVVSAAPTASRVSDLVKKSSSTCTFTSASEASESISSCSNVVLSNIE
                 ************************************************************

Endo_WT          VPAGETLDLSDAADGATITFEGTTSFGYEEWDGPLIRFGGKQLTITQSDGAVIDGDGSRW
Endo_SF          VPAGETLDLSDAADGATITFEGTTSFGYEEWDGPLIRFGGKQLTITQSDGAVIDGDGSRW
Endo_QN          VPAGETLDLSDAADGATITFEGTTSFGYEEWDGPLIRFGGKQLTITQSDGAVIDGDGSRW
                 ************************************************************

Endo_WT          WDSEGTNGGKTKPKFMYVHDVEDSTIKGLQIKNTPVQAISVQATNVYLTDITIDNSDGDD
Endo_SF          WDSEGTNGGKTKPKFMYVHDVEDSTIKGLQIKNTPVQAISVQATNVYLTDITIDNSDGDD
Endo_QN          WDSEGTNGGKTKPKFMYVHDVEDSTIKGLQIKNTPVQAISVQATNVYLTDITIDNSDGDD
                 ************************************************************

Endo_WT          NGGHNTDGFDISESTGVYISGATVKNQDDCIAINSGENILFTGGTCSGGHGLSIGSVGGR
Endo_SF          NGGHNTDGFDISESTGVYISGATVKNQDDCIAINSGENILFTGGTCSGGHGLSIGSVGGR
Endo_QN          NGGHNTDGFDISESTGVYISGATVKNQDDCIAINSGENILFTGGTCSGGHGLSIGSVGGR
                 ************************************************************

Endo_WT          DDNTVKNVTISDSTVTDSANGVRIKTIYGDTGDVSEITYSNIQLSGITDYGIVIEQDYEN
Endo_SF          DDNTVKNVTISDSTVTDSANGVRIKTIYGDTGDVSEITYSNIQLSGITDYGIVIEQDYEN
Endo_QN          DDNTVKNVTISDSTVTDSANGVRIKTIYGDTGDVSEITYSNIQLSGITDYGIVIEQDYEN
                 ************************************************************

Endo_WT          GSPTGTPSTGVPITDVTVDGVTGSIEDDAVQVYILCGDGSCSDWTWSGVDITGGKTSSDC
Endo_SF          GSPTGTPSTGVPITDVTVDGVTGSIEDDAVQVYILCGDGSCSDWTWSGVDITGGKTSSDC
Endo_QN          GSPTGTPSTGVPITDVTVDGVTGSIEDDAVQVYILCGDGSCSDWTWSGVDITGGKTTSDC
                 ********************************************************:***

Endo_WT          ENVPSGASC
Endo_SF          ENVPSGASC
Endo_QN          ENVPSGASC
                 *********
```

Figure S3. Amino acid sequence alignment of putative endo-polygalacturonase gene from BCC60424 (WT), SF-034 double mutant (SF), and QN-247 quadruple mutant (QN) strains.

Putative exo-polygalacturonase

```
Exo_WT          MRTSHLLSQALGLLALGATVEGFNRDRNQACGPHKPFQPLPTSKSRSKTCHVRTHGDGSD
Exo_SF          MRTSHLLSQALGLLALGATVEGFNRDRNQACGPHKPFQPLPTSKSRSKTCHVRTHGDGSD
Exo_QN          MRTSHLLSQALGLLALGATVEGFNRDRNQACGPHKPFQPLPTSKSRSKTCHVRTHGDGSD
                ************************************************************

Exo_WT          DSAYILSAIKQCNNGGKVVFDADKEYIIGTALDLTFLKHIDLDIEGTIQFTNDTDYWQAN
Exo_SF          DSAYILSAIKQCNNGGKVVFDADKEYIIGTALDLTFLKHIDLDIEGTIQFTNDTDYWQAN
Exo_QN          DSAYILSAIKQCNNGGKVVFDADKEYIIGTALDLTFLKHIDLDIEGTIQFTNDTDYWQAN
                ************************************************************

Exo_WT          AFQQVFQNATTFFQLGGEDVNVYGGGTLDGNGQVWYDLYAEDALILRPILFGIIGLNGGT
Exo_SF          AFQQVFQNATTFFQLGGEDVNVYGGGTLDGNGQVWYDLYAEDALILRPILFGIIGLNGGT
Exo_QN          AFQQVFQNATTFFQLGGEDVNVYGGGTLDGNGQVWYDLYAEDALILRPILFGIIGLNGGT
                ************************************************************

Exo_WT          IGPLNLRYSPQWYNLIANSTNVLYDGINISGYSKNPSVTAKNTDGRDTYRSSNIVIQNSV
Exo_SF          IGPLNLRYSPQWYNLIANSTNVLYDGINISGYSKNPSVTAKNTDGRDTYRSSNIVIQNSV
Exo_QN          IGPLNLRYSPQWYNLIANSTNVLYDGINISGYSKNPSVTAKNTDGRDTYRSSNIVIQNSV
                ************************************************************

Exo_WT          INNGDDCVSFKPNSTDILVQNLHCNGSHGISVGSLGQYLGEVDIVQDILVYNISMYNASD
Exo_SF          INNGDDCVSFKPNSTDILVQNLHCNGSHGISVGSLGQYLGEVDIVQDILVYNISMYNASD
Exo_QN          INNGEDCVSFKPNSTDILVQNLHCNGSHGISVGSLGQYLGEVDIVQDILVYNISMYNASD
                ****:*******************************************************

Exo_WT          MARIKVWPGVSSALSEDLQGGGGSGRVQNVTYDTAYIDNVDWAIEVTQCYGQKNLTLCNE
Exo_SF          MARIKVWPGVSSALSEDLQGGGGSGRVQNVTYDTAYIDNVDWAIEVTQCYGQKNLTLCNE
Exo_QN          MARIKVWPGVSSALSEDLQGGGGSGRVQNVTYDTAYIDNVDWAIEVTQCYGQKNLTLCNE
                ************************************************************

Exo_WT          YPSSLTISDVWFKNFRGKTSGKENPYVGTLVCSSPEVCSNIYTDNINVVSPKGTNDFVCD
Exo_SF          YPSSLTISDVWFKNFRGKTSGKENPYVGTLVCSSPEVCSNIYTDNINVVSPKGTNDFVCD
Exo_QN          YPSSLTISDVWFKNFRGKTSGKENPYVGTLVCSSPEVCSNIYTDNINVVSPKGTNDFVCD
                ************************************************************

Exo_WT          NVDTSDLSVNCTATSS
Exo_SF          NVDTSDLSVNCTATSS
Exo_QN          NVDTSDLSVNCTATSS
                ****************
```

Figure S4. Amino acid sequence alignment of putative exo-polygalacturonase gene from BCC60424 (WT), SF-034 double mutant (SF), and QN-247 quadruple mutant (QN) strains.

Table S1. Chemical compositions of coffee by-products.

Component	Coffee pulp (CP)	Coffee husk (CH)	Coffee silverskin (CS)	Spent coffee ground (SCG)
Carbohydrate	57	58-85	62-67	71-82
Cellulose	10-36	23-35	18-23.8	12.40
Hemicellulose	9.2-29	13-30	13-16.7	39.10
Lignin	26-31.5	23-24.5	28.6	24
Pectin	20.5	6.5	ND	ND
Reference	Frómeta et al., 2020[1]; Tripathia and Murthy, 2023[2]	Gouvea et al. 2009[3]; Tripathia and Murthy, 2023	Tripathia and Murthy, 2023	Tripathia and Murthy, 2023

[1] Frómeta RAR, Sánchez JL, García JMR. 2020. Evaluation of coffee pulp as substrate for polygalacturonase production in solid state fermentation. *Emir J Food Agric* 117.
[2] Tripathi S, Murthy PS. 2023. Coffee oligosaccharides and their role in health and wellness. *Food Res Int.* **173**:113288.
[3] Gouvea BM, Torres C, Franca AS, Oliveira LS, Oliveira ES. 2009. Feasibility of ethanol production from coffee husks. *Biotechnol Lett.* **31**:1315-9.

Table S2. Enzyme activity profile of crude enzyme preparations from *A. aculeatinus* SF-034 and QN-247 mutants.

Enzyme activity[1]	Volumetric activity (U/mL)			Specific activity (U/mg protein)		
	BCC60424 (WT)	SF-034	QN-247	BCC60424 (WT)	SF-034	QN-247
Pectinase	70.20 ± 0.30	114.20 ± 0.37	157.25 ± 1.53	96.25 ± 0.32	117.76 ± 0.27	98.90 ± 0.96
Endo-polygalacturonase	ND	154.96 ± 0.94	369.90 ± 0.47	ND	166.62 ± 1.01	232.33 ± 0.29
Endoglucanase (CMCase)	48.16 ± 0.07	79.10 ± 0.02	97.14 ± 1.63	65.94 ± 0.10	81.57 ± 0.05	61.00 ± 1.03
Exoglucanase (FPase)	0.61 ± 0.07	4.21 ± 0.13	3.90 ± 0.03	0.85 ± 0.11	4.34 ± 0.15	2.45 ± 0.02
β-glucosidase	147.45 ± 7.32	748.61 ± 1.29	601.58 ± 11.85	204.22 ± 10.64	774.55 ± 10.68	377.84 ± 7.45
Xylanase	42.51 ± 0.21	95.02 ± 0.36	53.45 ± 7.20	58.26 ± 0.15	97.96 ± 0.24	33.57 ± 2.61
Mannanase	16.61 ± 0.19	18.78 ± 0.13	12.23 ± 0.76	22.76 ± 0.17	19.36 ± 0.11	7.68 ± 0.47
β-glucanase	265.15 ± 0.03	577.15 ± 0.06	412.47 ± 5.44	363.22 ± 0.05	595.10 ± 0.04	259.07 ± 3.42
Amylase	4.35 ± 0.01	4.35 ± 0.01	3.51 ± 0.20	6.47 ± 0.11	4.49 ± 0.06	2.02 ± 0.12
Total protein (g/L)	0.73 ± 0.01	0.97 ± 0.02	1.59 ± 0.03	-	-	-
Source	[13]	[13]	This study	[13]	[13]	This study

[1] Enzyme activity of each enzyme was determined following the previously described method [13].
